# Discussing Sexual Health in the Medical Oncologist’s Practice: Exploring Current Practice and Challenges

**DOI:** 10.1007/s13187-019-01559-6

**Published:** 2019-06-17

**Authors:** E. M. Krouwel, L. F. Albers, M. P. J. Nicolai, H. Putter, S. Osanto, R. C. M. Pelger, H. W. Elzevier

**Affiliations:** 1 Department of Urology, J3P, Leiden University Medical Centre, PO Box 9600, 2300 WB Leiden, The Netherlands; 2 Department of Medical Decision Making, Leiden University Medical Centre, PO Box 9600, 2300 WB Leiden, The Netherlands; 3 Department of Medical Statistics, Leiden University Medical Centre, PO Box 9600, 2300 WB Leiden, The Netherlands; 4 Department of Oncology, Leiden University Medical Centre, PO Box 9600, 2300 WB Leiden, The Netherlands

**Keywords:** Sexual health care, Oncology, Quality-of-life, Communication, Sexual dysfunction

## Abstract

Sexuality is a significant quality-of-life concern for many cancer patients. Patients may be disadvantaged if they are not informed and not offered sexual health care. We sought to reveal oncologists’ current practice and opinions concerning sexual counselling. The aim of this study was to explore the knowledge, attitude and practice patterns of Dutch medical oncologists regarding treatment-related sexual dysfunction. Questionnaires were sent to 433 members of the Dutch Society of Medical Oncology. The majority (81.5%) of the 120 responding medical oncologists (response rate 30.6%) stated they discussed sexual function with fewer than half of their patients. At the same time, 75.8% of the participating oncologists agreed that addressing sexual function is their responsibility. Sexual function was discussed more often with younger patients and patients with a curative treatment intent. Barriers for avoiding discussing sexual function were lack of time (56.1%), training (49.5%) and advanced age of the patient (50.4%). More than half (64.6%) stated they had little knowledge about the subject and the majority (72.9%) wanted to acquire additional training in sexual function counselling. Medical oncologists accept that sexual function counselling falls within their profession, yet they admit to not counselling patients routinely concerning sexual function. Only in a minority of cases do medical oncologists inform their patients about sexual side effects of treatment. Whether they counsel patients is related to how they view patient’s prognosis, patient’s age, and self-reported knowledge. Findings indicate there is a role for developing education and practical training.

## Introduction

It is widely known that sexual dysfunction is a common side effect of oncological disease. All cancer therapies, including chemo-, hormonal- and immunotherapy, radiation and surgery can impair the sexual function. The prevalence of sexual side effects following therapy varies, depending on cancer and therapy type, but may even rise to 100% after treatment of genital cancers [1–5]. Cancer patients often face sexual symptoms from the start of treatment and these are likely to continue or even increase in the long term [6]. The consequences of cancer treatment can influence all aspects of sexuality, including desire, satisfaction and functioning. Sexuality is considered an extremely important quality-of-life concern by cancer survivors [7–9]. Despite reporting concerns regarding their sexual function, patients are frequently not informed about how treatment may affect their sexual function [1, 10, 11].

Given the high prevalence of sexual dysfunction and the complexity of the problems, an integrative approach to potential sexual problems is needed. Literature reveals a mismatch in expectations between the patient and healthcare providers regarding communication about sexuality [12–14]. Patients reported unmet needs regarding discussing sexuality with their health care providers. While some patients wish to discuss this topic, they feel health care providers do not provide an opportunity to talk about sexual function or even ignore their sexual needs [5, 11, 12, 15–17]. On the other hand, not all healthcare professionals consider it their task to discuss the subject [18]. Moreover, they face several other barriers, such as uncomfortable feelings, insufficient knowledge, lack of training, lack of time and over involvement in aspects of patients’ personal lives. Oncology care providers do, however, consider sexual function to be an important topic [18–21]. During cancer treatment, patients are treated by different professionals within a multidisciplinary team. It is not always clear which member of the team is responsible for addressing sexual function. Studies among different Dutch oncology care providers revealed that members of the oncology team, like radiation oncologists, oncology nurses and oncology surgeons, see some role for themselves in sexual function counselling, but all point to the medical oncologist to bring up the subject [19–21].

Consequently, it is important to identify how medical oncologists report their own role in sexual counselling. An understanding of how medical oncologists acquire knowledge about sexual function counselling, how they apply sexual function counselling in practice, and which barriers they may encounter when bringing up the subject is needed to optimise management around sexual care for oncology patients. The aim of this study is to explore the attitude, practice patterns and education needs of medical oncologists regarding sexual function counselling.

## Methods

### Study Design

A questionnaire was used to collect data in a cross-sectional survey. The questionnaire was sent to 433 members of the NVMO (Dutch Society of Medical Oncology). The total number was 440, but 7 members living and practising oncology abroad were excluded (most of them from the Netherlands Antilles). Members of the NVMO include both medical oncologists and oncology differentiating residents. Our sampling strategy aimed to represent area of expertise, employment setting, level of education, years of oncology experience, type of hospital, age and gender.

### Survey Administration

The questionnaires and reminders were sent in 2014. Non-responders received a reminder twice. The questionnaires were sent by post and included a stamped, addressed envelope. Reason for using a postal survey was to obtain the highest possible response rate. In studies with participants between 30 and 60 years old or older, the highest response rate was seen in postal surveys [22–24]. We expected the average age of our respondents to be older than 30 years. Furthermore, we wanted to prevent younger, male, avid Internet users and those with greater technological interest to be over-represented in the survey [22, 25].

### Instrument Design and Development

The questionnaire consisted of 38 questions (Appendix 1). It contained questions on demographics, frequency of discussing sexual function, the patient’s view about the responsibility for discussing sexual function, barriers faced when discussing sexual function, self-reported knowledge about sexual function after cancer treatment and the need for additional training. The questionnaire was developed by the authors based on several items found in relevant literature and on previously conducted sexuality questionnaire studies among health care professionals. The latter was derived from our research group and concerned questions about practice patterns, knowledge, barriers and responsibility regarding treatment-related sexual function [19–21]. The content of the questionnaire was pilot-tested by four oncologists from the area of Leiden, the Netherlands. A small pilot panel was chosen because of the limited number of oncologists in the Netherlands; the members of the pilot panel were not invited for the survey. The pilot panel reviewed the questionnaire with regard to relevance, integrity, structure, layout and spelling.

### Analysis

Data analysis was performed using SPSS (Release 23; SPSS Inc.). Demographic information and answers to the survey were analysed using descriptive statistics. Equality of proportions between groups was tested with Pearson’s chi-square test; for ordinal variables, the Armitage’s trend test was applied. Continuous variables were compared using Student’s *t* test. Age groups were divided into two groups: under 47 years and 47 years and older (according to median age of 47 years). The group was divided into two according to experience: up to 10 years and more than 10 years of experience. Two-sided *P* values < .05 were considered statistically significant.

### Ethical Consideration

The study was formally approved by the scientific committee of the Department of Urology of the LUMC. In the Netherlands, research that does not involve patients or interventions is not subject to permission from ethical boards. In previous research using similar types of questionnaires, the Medical Ethics Committee was consulted by our research group. As the study did not concern information recorded by the investigator in such a manner that subjects could be identified, and as it did not compromise the study participants’ integrity, the Committee declared that no formal ethical approval was needed.

## Results

### Participants

The survey was distributed among 433 medical oncologists; 209 of them responded (initial response rate 48.3%). Of these 209 responders, nine were returned to sender, 26 oncologists reported they had retired and 6 were not medical oncologists. A notification of refusal was received from 48, 39.3% (*n* = 35) of whom refused due to lack of time. Of 392 eligible participants, 120 completed questionnaires were returned and included for analysis, resulting in a final response rate of 30.6%.

The mean age of the respondents was 47 years (range 30–64) and half of them (*n* = 56 52.5%) were male. The male respondents were significantly older than female respondents (*p* < 0.001). The majority (*n* = 72, 61%) reported > 5 years of experience working in the field of oncology. Areas of expertise and clinical settings are presented in Table 1.

**Table 1:** Participant characteristics

Oncologists (*n* = 120)	
Median age in years (range) Age of male respondents (years) Age of female respondents (years)	47 (30–64) 50.6 (SD 10) 41.9 (SD 8.9)
Gender	*n* (%)
Male	56 (46.7)
Female	63 (52.5)
Unknown	1 (0.8)
Function	
Oncologist	101 (84.2)
Oncology resident	19 (15.8)
Area of expertise^a^	
Breast	88 (73.3)
Colorectal	79 (65.8)
Palliative care	57 (47.5)
Gynaecology	53 (44.2)
Nephrology and urology	53 (44.2)
Haematology	37 (30.8)
Lymphoma	32 (26.7)
Head and neck	14 (11.7)
Neuroendocrine	14 (11.7)
Melanoma	8 (6.7)
Sarcomas	8 (6.7)
Lung	3 (2.5)
Type of practice	
District general hospital	47 (39.2)
University hospital	40 (33.3)
District general teaching hospital	27 (22.5)
Cancer institute	3 (2.5)
Both university and district	2 (1.7)
Unknown	1 (0.8)
Oncology experience	
< 1 year	0
1–2 years	19 (15.8)
3–5 years	27 (22.5)
6–10 years	13 (10.8)
11–15 years	19 (15.8)
> 15 years	40 (33.3)
Unknown	2 (1.7)

^a^Most respondents reported multiple areas of expertise

### Addressing Sexuality in Medical Practice

The medical oncologists participating in this survey estimated that 70.6% (SD 17.21, range 20–100%) of their patients may experience sexual changes as a result of cancer treatment. Most respondents (*n* = 97, 81.5%) reported discussing sexual function in fewer than 50% of their patients. There was no significant difference in frequency of discussing sexual function between male and female specialists, years of experience or age of the oncologist (resp. *p* = 0.503, *p* = 0.471, *p* = 0.178). Three-quarters (*n* = 90) of the responding oncologists stated that they discussed sexual function in fewer than half of the cases during the informed consent conversation before the start of treatment. Findings are summarized in Table 2. The main topics being discussed were decreased libido (*n* = 65, 72.2%), menopausal symptoms (*n* = 63, 70%), insufficient lubrication (*n* = 60, 66.7%) and pain during intercourse (*n* = 48, 53.3%) in women. Erectile dysfunction (*n* = 74, 82.2%) and decreased libido (*n* = 73, 81.1%) were frequently discussed with male patients.

**Table 2:** Discussing sexual function in daily practice

	Total respondents	Never/rarely *n* (%)	In fewer than half of the cases *n* (%)	In half of the cases *n* (%)	In more than half of the cases *n* (%)	Often/always *n* (%)
How often do you discuss sexual function with your patients?	118	43 (36.1)	54 (45.4)	16 (13.4)	3 (2.5)	2 (2.5)
How often do you inform your patients about the possible effects on sexual health during an informed-consent conversation?	120	37 (30.8)	53 (44.2)	14 (11.7)	10 (8.3)	6 (5)
During follow-up, how often do you discuss sexual health with patients?	90	37 (40.7)	45 (49.5)	3 (3.3)	5 (5.5)	0

**Fig. 1:**
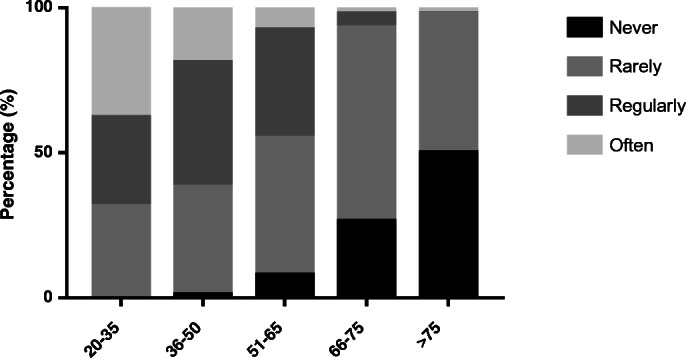
How often do you discuss sexuality with in the following age groups (years)?

Among oncologists who did discuss sexual function, 91.4% (*n* = 83) reported addressing this subject when treatment had a curative intent. This declined to 62.4% (*n* = 57) when the treatment had a life-prolonging intent and to 33.3% (*n* = 30) in cases of palliative treatment. The oncologists discussed sexuality more often with younger patients. Sixty-eight per cent (*n* = 61) of the respondents discussed sexuality regularly/always with patients between 20 and 35 years of age; this percentage declined to 2.2% (*n* = 2) in patients older than 75 years. All age groups are represented in Fig. 1.

### Responsibility and Barriers

Of all oncologists, a large majority of 75.8% (*n* = 91) stated they felt responsible for discussing sexual function with their patients. A similar percentage (75%, *n* = 90) indicated they considered the oncology nurse also to be responsible and half of the respondents (*n* = 61) thought the patient was responsible for initiating the subject. Responsibility allocated to possible health care providers and the patient or partner is displayed in Table 3. A minority (*n* = 14, 12.8%) of respondents stated there was an agreement defining responsibility for discussing sexual function within their multidisciplinary team.

**Table 3:** Responsibility for addressing sexual health according to the oncologists

Who is responsible for addressing sexual function? (multiple answers possible)	*n* (%)
Oncologist	91 (75.8)
Oncology nurse	90 (75)
Patient	61 (50.8)
Partner of patient	28 (23.3)
General practitioner	28 (23.3)
Psychologist	14 (11.7)
Social worker	6 (5)
Physiotherapist	1 (0.8)

According to the medical oncologists, the major barriers for discussing sexual function were ‘lack of time’ (*n* = 64, 56.1%), ‘advanced age of the patient’ (*n* = 57, 50.4%), ‘lack of training’ (*n* = 51, 49.5%) and ‘patient is too ill’ (*n* = 51, 49.5%). Less-experienced oncologists (≤ 10 years of practice) stated lack of time as a reason more often than their more experienced colleagues (*p* = 0.006). Other barriers to avoid having to address sexual function are listed in Table 4.

**Table 4:** List of boundaries for discussing sexual function

Reasons for avoiding discussion of sexual health	Total respondents*	Agree^a^ (%)	Partly agree/disagree	Disagree^a^ *n* (%)
Lack of time	114	64 (56.1)	27 (23.7)	23 (20.2)
Advanced age of the patient	113	57 (50.4)	26 (23)	30 (26.5)
Lack of training	113	51 (49.5)	35 (31.0)	27 (23.9)
Patient is too ill	114	51 (44.6)	35 (30.7)	28 (24.6)
No angle or motive for asking	114	45 (39.5)	39 (34.2)	30 (26.3)
Lack of knowledge	114	41 (36)	40 (35.1)	33 (28.9)
Patient does not bring up the subject	114	38 (33.3)	32 (28.1)	44 (38.6)
Culture/religion	114	27 (23.7)	34 (29.8)	53 (52.6)
Language/ethnicity	113	27 (23.9)	28 (24.8)	58 (51.3)
Surviving is more important	115	26 (23.1)	37 (32.7)	50 (44.2)
I feel uncomfortable	115	26 (22.8)	37 (32.5)	51 (44.7)
Sexuality is not a matter of life or death	114	25 (21.9)	37 (32.5)	52 (45.7)
Not relevant for all types of cancer	114	25 (21.9)	23 (20.2)	66 (57.9)
Presence of a third party	111	24 (21.6)	26 (23.4)	61 (54.9)
Patient is not ready to discuss sexual health	102	22 (19.7)	34 (30.4)	46 (50)
Sexuality is a private matter	131	21 (18.6)	53 (31)	57 (50.5)
Embarrassment	114	20 (17.6)	32 (28.1)	62 (62.3)
It is someone else’s task	113	17 (15)	27 (23.9)	69 (61)
No trust in treatment for sexual dysfunction	112	13 (11.6)	32 (28.6)	67 (59.8)
Concerned about causing the patient discomfort	114	12 (10.5)	30 (26.3)	72 (63.1)
Sexuality is not a patient’s concern	114	11 (9.7)	37 (32.5)	66 (57.9)
Age difference between you and patient	114	10 (8.8)	21 (18.4)	83 (72.8)
Afraid to offend the patient	114	6 (5.3)	15 (13.2)	93 (81.5)
Patient is the opposite gender	114	4 (3.5)	16 (14)	94 (82.4)
Patient is the same gender	112	0 (0)	7 (6.3)	105 (93.7)
Colleagues think it is inappropriate to discuss sexual issues with patients	113	0 (0)	11 (9.7)	102 (90.3)

*Not all respondents answered each question

### Knowledge, Education and Training Needs

A small percentage of the respondents (*n* = 14, 15.4%) stated they had sufficient knowledge to be able to discuss the subject. All other respondents (*n* = 77, 84.6%) stated having little or no knowledge of the subject. Oncologists with more self-stated knowledge discussed sexual function more often (*p* = 0.002). According to 85% (*n* = 102), education about sexual function counselling within their oncological training was insufficient. A majority of 72.9% (*n* = 86) would like to acquire more training in the counselling of sexual function, regardless of their self-stated knowledge (*p* = 0.733). No significant differences were found in training needs between areas of expertise.

## Discussion

The present study provides insight into the practice patterns of Dutch medical oncologists with regard to discussing sexual function. It reveals the origins of several difficulties in discussing sexual function in current clinical practice. Medical oncologists do see sexual function counselling as part of their duty. Nevertheless, they do not routinely counsel sexual function due to several barriers, such as lack of training. A minority informs their patients about potential sexual side effects of planned cancer treatment. Whether oncologists counsel patients is related to the age of the patient, how they view the patient’s prognosis and to whether they stated they had more knowledge about sexual function.

The results of this study are in line with other self-reported surveys among oncology health care providers about communication regarding sexual concerns. To our knowledge, this is the first study to describe how medical oncologists see their role in sexual counselling, depicting the actual origin of difficulties in discussing sexual issues in current clinical practice.

According to our data, Dutch oncologists rarely bring up sexual side effects during the informed consent conversation before starting a treatment. Informed consent is seen as a crucial component of medical practice and authenticates patients’ autonomy. During informed consent, adverse effects that are common should be discussed [26]. Given the high prevalence and additional burden of sexual dysfunction after cancer treatment, sexual side effects of treatment should be part of informed consent [1–5, 26]. Lack of knowledge, lack of time and lack of clarity about sexual side effects in current guidelines may result in ambiguity regarding responsibility for discussing sexual side effects [18]. An example of how to enhance communication about sexual side effects during informed consent is the use of an informed consent template, provided by the ASCO, where side effects, including sexual side effects are mentioned [27]. Nevertheless, a form cannot replace direct patient-provider communication but could help the care provider to address the subject.

Since sexual problems can arise during early treatment, but may also arise after treatment and even extend long term, discussing sexual function during the whole cancer care process would seem to be important [6]. However, the current survey revealed that Dutch oncologists do not routinely bring up the subject of sexuality during treatment and follow-up. According to the literature, other members of the multidisciplinary oncological team identified discussing sexual function as a responsibility of the oncologist [19–21]. Members of the multidisciplinary oncological team seem to count on each other to tackle the conversation about sexual health. This highlights the importance of defining responsibilities within the oncology treatment team. According to this survey, only 12.8% of the respondents reported a clearly defined responsibility for addressing sexuality within their team. De Vocht et al. described a Stepped-Skills-model, which could be of help to define responsibilities [18]. In this team-approach-model, there are team members who are ‘spotters’. These spotters, most likely the oncologist, discuss the sexual side effects of treatment, check whether patients need help and refer them where necessary. Other members, most probably the specialized nurses, are called ‘skilled companions’. They have the responsibility to support patients in their sexuality issues. Consequently, these members require training to improve their communication skills and their knowledge. Using such an integrated approach, sexual health may become part of daily clinical practice.

As already highlighted in the ‘Introduction’ section, a mismatch in expectations regarding the discussion of sexual health between patient and providers does exist. The current study reveals some of the reasons why medical oncologists do not bring up sexuality, which may contribute to this mismatch. Of the respondents, almost 60% stated the ‘advanced age of the patient’ as a barrier to discussing sexual function, suggesting respondents may assume elderly patients are not sexually active. This may be an incorrect assumption. A study on the prevalence of sexual activity among 10,000 European adults showed that sexual desire and activity persist through old age, with 53% of the male respondents and 21% of the female respondents between 70 and 80 years of age being sexually active [28].

Another barrier to discussing sexuality mentioned by almost half of the oncologists involved ‘the patient being too ill’. Also, frequency of bringing up sexual health declined when treatment had a palliative intent compared to a curative intent. A study reviewing sexual healthcare for cancer patients receiving palliative care confirmed a lack of sexual health care in this patient group, although the patients and their partners did feel the need for a conversation about the subject. Bringing up the subject of sexuality by a healthcare professional even improved quality of life and reduced stress of patients and partners [29]. An interdisciplinary approach is required to recognise and manage symptoms in this palliative group.

In accordance with previous investigations, important reasons for the lack of frequency in discussing sexual health were a ‘lack of training’ and a ‘lack of knowledge’ [15, 19–21]. These evidently recurrent barriers among different cancer care providers in different countries indicate that there is a role for education and practical training to improve the situation in practice. A pilot study involving 82 oncology providers showed that a brief (30–34 min) targeted sexual health training significantly enhanced the frequency of discussing sexual issues with cancer patients [30]. In Iceland, a sexual health care educational intervention was implemented over a 2-year time period. Over 200 oncology nurses and physicians participated. The study showed that the perceived level of knowledge in providing sexual health care was higher after the intervention [31]. Furthermore, communication tools, using standard patient questionnaires on sexuality resulted in improved communication between the patient and the health care provider regarding sexual function [32]. However, with the increasing pressure on daily practice of physicians and nurses, and taking another major barrier—lack of time—into consideration, we are urged to look for additional ways of providing sexual health care. Possibilities for educating patient and partner regarding sexual function during and after cancer treatment, like e-health, using websites, videos and apps, have to be further investigated and evaluated.

Some limitations need to be considered. As no validated questionnaires were available, a non-validated questionnaire was administered. The use of a self-reported questionnaire may have led to under- or overestimation. Questionnaire-based studies are always subjected to response and selection bias. A sampling error may have occurred due to the low response rate, although this rate was comparable to that found by other questionnaire studies. There may be a difference between the oncologists who responded and those who did not respond to our questionnaire, possibly creating a bias. The fact that a postal survey was used may have resulted in incomplete responses. Internet questionnaires are known to have a higher degree of completeness since the researcher is able to compensate for errors among respondents who for example accidentally pass over a question [24]. The subdivisions by area of specialization resulted in small numbers of medical oncologists in each group. For this reason, it was not possible to do proper sub-analyses per area. The area of specialisation of the majority of the responding oncologists was breast cancer. The questionnaire may, therefore, have been answered in the context of breast cancer, meaning the patients were slightly younger and were receiving (neo) adjuvant chemotherapy or hormonal therapy, with the accompanying effects on sexual functioning. A larger study among medical oncologists from different countries might be useful in defining differences between areas of specialisation.

The results of this study may improve the awareness of health care professionals in cancer treatment, especially medical oncologists, about the need to define the place of sexual health care in the course of the disease trajectory, to discuss if a specific team member is responsible for initiating the subject and, if necessary, provide additional training.

## Conclusion

The current study reveals that medical oncologists do not routinely counsel patients concerning sexual function being confronted by several barriers, although they do see this as part of their role. Patients’ prognosis, patients’ age and how knowledgeable the oncologist is about sexual function influence the frequency of counselling. Our findings indicate that there is a role for education and practical training for improving sexual health care in the oncology practice.
